# Breast Cancer in Africa: Limitations and Opportunities for Application of Genomic Medicine

**DOI:** 10.1155/2016/4792865

**Published:** 2016-06-16

**Authors:** Allison Silverstein, Rachita Sood, Ainhoa Costas-Chavarri

**Affiliations:** ^1^Department of Plastic and Oral Surgery, Boston Children's Hospital, Boston, MA 02115, USA; ^2^Harvard Medical School, Program in Global Surgery and Social Change, Boston, MA 02115, USA; ^3^University of Miami Miller School of Medicine, Miami, FL 33136, USA; ^4^Rwanda Military Hospital, Kigali, Rwanda

## Abstract

As genomic medicine gains clinical applicability across a spectrum of diseases, insufficient application in low-income settings stands to increase health disparity. Breast cancer screening, diagnosis, and treatment have benefited greatly from genomic medicine in high-income settings. As breast cancer is a leading cause of both cancer incidence and mortality in Africa, attention and resources must be applied to research and clinical initiatives to integrate genomic medicine into breast cancer care. In terms of research, there is a paucity of investigations into genetic determinants of breast cancer specific to African populations, despite consensus in the literature that predisposition and susceptibility genes vary between populations. Therefore, we need targeted strengthening of existing research efforts and support of new initiatives. Results will improve clinical care through screening and diagnosis with genetic testing specific to breast cancer in African populations. Clinically, genomic medicine can provide information capable of improving resource allocation to the population which most stands to benefit from increased screening or tailored treatment modalities. In situations where mammography or chemotherapy options are limited, this information will allow for the greatest impact. Implementation of genomic medicine will face numerous systemic barriers but is essential to improve breast cancer outcomes and survival.

## 1. Introduction

The clinical spectrum of breast cancer management, from screening to diagnosis to treatment, benefits widely from applications of genomic medicine in high-income countries (HICs). With genomic medicine rapidly emerging as a means for personalized and improved medical care, attention must be paid to research and integration in low-income settings. Otherwise, disparate implementation of genomic technology will continue to heighten existing health disparities [1, 2].

Women who present to their gynecologists, surgeons, and other healthcare providers in HICs may undergo genetic testing to evaluate the presence of specific mutations linked to increased breast cancer risk. These results not only guide screening decisions, but also have the potential to influence prophylactic medical and surgical treatment options. Further, upon diagnosis of breast cancer, immunohistochemistry studies can identify genetic markers to characterize the tumor and enable targeted treatment. Patients may elect to use broader genetic panels, such as Oncotype DX, which help determine risk of recurrence to further tailor treatment recommendations [3–5].

The Global Burden of Cancer 2013 asserted that breast cancer is a leading cause of cancer death in Africa and has the highest incidence of all cancers [6]. Although breast cancer is the most common cancer in women worldwide, case fatality rates are highest in low- and middle-income countries (LMICs) [7, 8]. We argue that research and implementation efforts must be undertaken to similarly integrate genomic medicine into breast cancer care in Africa and that doing so has tremendous potential to improve outcomes and survival. The need for continent-wide focus on improving breast cancer outcomes is clear.

## 2. A Research Agenda for Africa

There is a paucity of well-powered research investigating the possibility of genetic predisposition to breast cancer in Africa [9, 10]. This lack of research impairs our ability to fully understand disease presentation and progression. Studies suggest that hereditary mutations are responsible for up to 5% of patients presenting with breast cancer [10]. While novel BRCA mutations have been discovered in Afrikaner populations [11], we must work on creating and increasing the existence of broad breast cancer genetic registries specific to and with recognition of the variability in the African population. A 2012 systematic literature review found that there was less than one study per year in the African population since the discovery of the BRCA gene; this is far from sufficient. Further, studies that have discovered new germ line mutations have been underpowered to make generalizations about the population [10]. In the African population where tumor characteristics appear to be different, we must uncover new genetic patterns and utilize our findings to diagnose cases earlier and improve care.

Disparities in racial representation in research are not limited simply to breast cancer; despite knowledge that genetic variants have different effects in different ethnic groups, 96% of subjects included in genome-wide association studies (GWAS) are of European descent [12]. Additionally, African populations demonstrate more genetic diversity than on other continents and require differentiation of subpopulations in investigating genetic determinants of health [13].

Initiatives such as the Human Heredity and Health (H3) Network aim to facilitate research about genomics and environmental determinants of common diseases to improve the health of Africans. Among a number of ongoing projects is the H3Africa Kidney Disease Research Network, which studies prevalent forms of kidney disease in sub-Saharan Africa. In addition to the collection and analysis of genetic information, they strive to build research capacity by training relevant personnel and developing facilities that can support genetic analyses and storage of information in Africa [14]. While this strategy will help bridge the existing divide for H3's targeted diseases and ultimately have broader implications, we maintain that a greater focus must be placed on breast cancer at this time.

## 3. Genomic Medicine in Clinical Care

Expansion of genomic medicine research specific to African populations will generate knowledge for future innovation and ultimately enable clinical applications. An understanding of current applications of and limitations to the integration of genomic medicine into breast cancer screening and care in Africa is essential to designing an effective plan for the future. Knowledge of genetic predisposition can alter patients' treatment decisions before they even develop cancer; in high-resource settings, BRCA+ mutation carriers may elect to undergo prophylactic mastectomy or oophorectomy or opt for nonsurgical methods of risk reduction [15, 16]. Ultimately, this can help reduce the cost and discomfort associated with recurrent surgeries. In South Africa, for example, where genetic testing for BRCA mutations has been available since 2005, in order to conserve resources, women affected with breast cancer are first tested instead of the “worried well” [16]. In addition to integrating BRCA1/2 genetic testing, if we can identify novel genetic mutations, we can combine this information to assist families of patients by testing them for the same mutations and guiding them to personalize their screening and treatment options.

However, given the financial and resource limitations to universally available genetic testing in low-income health systems, a simple family history can serve as a marker for increased breast cancer risk [17, 18]. A case-control study in Nigeria evaluating 250 women with breast cancer and 250 age-matched controls found that family history of breast cancer was associated with a 15-fold increase of breast cancer [17]. There are multiple risk stratification tools that consider parameters, including family history of disease, age, parity, history of breastfeeding, and other demographic or social factors that may be easily asked of patients. These tools are used in HICs to adjust recommended frequency and type of screening and could be implemented at low cost and resource use in African countries.

Unfortunately, even if such scoring systems were utilized in said settings, many hospitals and countries face severe limitations in availability and utilization of screening and diagnostic services. A 2013 study in Nigeria investigating financial barriers to utilization of screening and treatment services of breast cancer noted that few centers have functional radiotherapy equipment and, where available, high costs limit widespread utilization [19]. Additional studies have also found direct links between poverty and low usage of mammography screenings [20]. The Breast Health Global Initiative guidelines recommend utilization of ultrasound in settings with limited resources and only recommend diagnostic mammography in settings where there are enhanced or maximal resources. However, they also acknowledge that screening mammography is the only single modality demonstrating improved breast cancer mortality in prospective randomized trials [21]. Integration of genomic medicine into screening plan design may help countries selectively choose how to best allocate their limited resources and would allow for broadening of screening services, specifically first to those most in need. As screening services continue to expand and are offered to the entirety of the country, countries could eventually employ risk stratification to alter the frequency of the services.

Breast cancer patients in Africa more commonly present at young ages and with advanced-stage disease [7, 22–24]. As a result, breast conservation surgery is often not an option and patients experience higher mortality rates [7]. Breast cancer mortality can most effectively be reduced via early detection and subsequent appropriate treatment—options that are more readily possible via integration of genomic medicine [24]. More so, advanced breast cancer requires more extensive utilization of resources; thus, efforts to identify cases early may also provide the most substantial benefit in terms of reducing cost [25]. While the mastectomy rate is 30% in Europe [26, 27], it is greater than 85% in some regions of Africa [28]. Although lack of screening, diagnostic capabilities, or social determinants may influence differences in breast cancer care among the African population, one must question whether variations in genetics influence this distinct, aggressive presentation of breast cancer and its outcomes [9].

One of the first applications of genomic medicine into breast cancer care in HICs was the identification of estrogen receptor (ER), progesterone receptor (PR), and human epidermal growth factor receptor 2 (HER2) tumor markers to target treatment. Unfortunately, receptor testing is not available or not routinely utilized in many low-resource settings, therefore inhibiting proper clinical distribution of endocrine therapy to those patients who will most likely respond [22, 29, 30]. Immunohistochemistry is arguably even more important in LMICs given the desire to avoid waste of limited resources on patients who will not respond to a specific treatment, for example, treating an ER negative patient with tamoxifen. Savings associated with selective use of hormonal therapy are greater than those costs associated with hormone-receptor testing [23, 25].

Studies have repeatedly revealed that African populations are more likely to possess triple negative tumors; these tumors are characterized by their aggressive nature and ineffective response to hormone therapy [23, 28, 31, 32]. While we do recommend continued testing to characterize tumors, we must also acknowledge the common tumor characteristics among the African population. Accordingly, we must work to identify new tumor markers and new therapies for triple negative tumors to optimally aid these populations.

## 4. Conclusion: A Call to Action

In the era of evolving medicine and advances in genomic medicine, limited applications of genomic medicine in LMICs further highlight the overall lack of equity in cancer care around the world. As money and time are invested in developing national cancer control plans and implementing screening guidelines, so too should attention be paid to genomic medicine and its opportunity to enhance cancer screening, diagnosis, and treatment. We need to recognize that genomic medicine is an emerging field in Africa and other LMICs and not just HICs.

Strengthening Africa's healthcare system in parallel with integrating genomic information will ultimately allow for the best care for patients. First, in generating and strengthening cancer control plans, countries must clinically integrate genomic medicine to best allocate already limited resources. Second, genetic variance must be considered amidst the African population and drive targeted research and screening. Implementing the above strategies will not be easy; however, success in doing so can drastically change the care and outcomes of future breast cancer patients.
